# Constipation in Chemotherapy Patients: A Diagnostic Accuracy Study

**DOI:** 10.31557/APJCP.2021.22.9.3017

**Published:** 2021-09

**Authors:** Isabel Neves Duarte Lisboa, Jéssica Dantas de Sá Tinôco, Dantas de Sá Tinôco2, Maria Isabel da Conceição Dias Fernandes, Rammila Rayara da Silva, Juliana Barbosa da Silva, Millena Freire Delgado, Marcos Venícios Oliveira Lopes, Ana Luisa Brandão de Carvalho Lira

**Affiliations:** 1 *Department of Nursing, Federal University of Rio Grande of Norte, Natal, Brazil. *; 2 *Department, State University of Rio Grande of Norte, Caicó, Brazil. *; 3 *Department of Nursing, Federal University of Ceará, Fortaleza, Brazil. *

**Keywords:** Drug therapy, constipation, data accuracy, nursing

## Abstract

**Objective:**

To analyze the accuracy of clinical indicators of constipation in cancer patients undergoing chemotherapy.

**Methods:**

A diagnostic accuracy study was conducted from February to September 2018, with 240 cancer patients undergoing chemotherapy. The data collection instrument was a form with sociodemographic and clinical variables, and clinical indicators of constipation. The sensitivity and specificity of the clinical indicators of interest were calculated using a latent class analysis approach.

**Result:**

The prevalence of constipation in the sample was 86.6%. The most sensitive clinical indicators were straining with defecation (100.0%), and hypoactive bowel sounds (75.0%), while headache (99.9%), abdominal pain (75.0%), pain with defecation (75.0%), straining with defecation (99.9%) and liquid stool (78.1%) were indicators with high specificity.

**Conclusion:**

A set of six clinical indicators was significantly associated with the occurrence of constipation in cancer patients undergoing chemotherapy, especially straining with defecation. These indicators can be used by nurses to identify constipation and propose prompt and effective interventions.

## Introduction

Cancer is the second-leading cause of death worldwide and affected approximately 9.6 million people in 2018 (Pan American Health Organization, 2019). Chemotherapy is the main treatment and corresponds to the administration of oral or intravenous cytotoxic agents, but its side effects are systemic (Brenner et al., 2017; Roy and Saikia, 2016). Chemotherapy may cause damage to enteric neurons and gastrointestinal dysfunction. Such damage persists for years after treatment, affecting patients’ quality of life (Brenner et al., 2017; Escalante et al., 2017). 

One of the common side effects of chemotherapy is constipation, a decrease in the normal frequency of defecation accompanied by difficult or incomplete passage of stool, or passage of excessively hard, dry stool (Herdman and Kamitsuru, 2018). Constipation is a common problem in cancer patients, with a prevalence of 40% to 90% (Larkin et al., 2018; Staats et al., 2004). The occurrence of this problem ranges from mild to severe and may cause depression, anxiety, nausea, vomiting, hemorrhoids, anal fissure, intestinal obstruction, and urinary retention (Larkin et al., 2018; Dhingra et al., 2013; Thorpe, 2001). Patients with untreated constipation incur expenses for the health system. Thus, accurate identification of constipation can contribute to early treatment and lower health costs (Larkin et al., 2018; Iskedjian et al., 2011; Wee et al., 2010).

Although the manifestations of constipation are commonly found in clinical practice (Agra et al., 2013; Brazil, 2009), the process by which nurses infer this problem is not always based on accurate clinical indicators, thus, it is of fundamental importance that nurses use accurate indicators in clinical practice to support the inference of constipation in cancer patients. The development of diagnostic accuracy studies on the sensitivity and specificity of specific clinical indicators is indispensable for the strengthening of nursing practice (Silva et al., 2017). 

Given the above, this study hypothesizes that there is a set of clinical indicators that best represent constipation in cancer patients undergoing chemotherapy. This study aimed to analyze the accuracy of clinical indicators of constipation in cancer patients undergoing chemotherapy.

## Materials and Methods

*Study design and setting*

A diagnostic accuracy study was carried out in a reference cancer service in Brazil. A prospective approach was used and the data collection was planned before the index test and reference standards were performed (Bossuyt et al., 2015). 

*Population*

The population consisted of patients undergoing chemotherapy at the service mentioned above. The sample size was estimated using the method described by Swanson et al., (2012) based on the Item Response Theory, recommended for diagnostic accuracy studies based on latent class analysis. In this method, the author proposes a predefined number of individuals per clinical indicator investigated. The sample calculation used a constant of nine for each of the 26 investigated clinical indicators. Thus, the sample was 240 patients.

A convenience, consecutive sampling method was used in this study. Patients with a medical diagnosis of cancer were identified by an oncologist specialist researcher using the database of the oncology hospital. Afterward, the following inclusion criteria were applied: age over 18 years, antineoplastic chemotherapy or chemotherapy combined with radiotherapy, and hormone therapy. The exclusion criteria were patients disoriented in time, place, and/or person, and patients undergoing chemotherapy for the first time (due to the absence of effects). 

*Data collection*

The data collection occurred from February to September 2018. The data collection instrument consisted of a form with sociodemographic variables (age, gender, marital status, education, personal and family income, and religion), clinical variables (treatment time, type of cancer, and medications used), and clinical indicators of constipation covered by the NANDA-I taxonomy of nursing diagnoses (Herdman and Kamitsuru, 2018) (abdominal pain, abdominal tenderness with palpable muscle resistance, abdominal tenderness without palpable muscle resistance, anorexia, atypical presentations in older adults, borborygmi, bright red blood with stool, change in bowel pattern, decrease in stool frequency, decrease in stool volume, distended abdomen, fatigue, hard formed stool, headache, hyperactive bowel sounds, hypoactive bowel sounds, indigestion, liquid stool, pain with defecation, palpable abdominal mass, percussed abdominal dullness, rectal fullness, rectal pressure, severe flatus, straining with defecation, and vomiting).

The main investigator used a standard operating protocol with operational definitions constructed for the identification of the clinical indicators of interest based on concepts from the Word Gastroenterology Organization (2010). 

Subsequently, the investigator assessed each clinical indicator and classified it as present or absent, except for hard formed stool, liquid stool, change in bowel pattern, decreased stool frequency, and anorexia, which were considered present or absent through measurement using validated tools.

The indicators hard formed stool and liquid stool were measured using the Bristol Scale (Lewis and Heaton, 1997), which allows the visual classification of stools into seven different types. The indicators change in bowel pattern and decrease in stool frequency were measured based on the criteria established in the Rome Consensus (Drossman, 2016). Anorexia was measured using the body mass index (Stengel et al., 2013).

*Data analysis*

Data were analyzed using the R software version 3.0.2. The descriptive analyses included frequencies, central tendency, and dispersion measures. The Kolmogorov-Smirnov test was used to determine normality.

Measures of specificity and sensitivity were obtained for each clinical indicator of constipation based on mathematical modeling, the latent class. This model indicates that unobservable or latent data determines the relationship between observable data (Collins and Lanza, 2010). In this study, the unobservable or latent data was constipation, and observable data corresponded to the clinical indicators investigated. Sensitivity is defined as the presence of a given clinical indicator when the outcome (constipation) is present; while specificity indicates the absence of a given indicator in the absence of the outcome (Lopes et al., 2012). 

This study used two latent class models with random effects to obtain sensitivity and specificity measures, and 95% confidence intervals. An initial null model contemplated all clinical indicators studied. Then, the likelihood ratio test (G2) was applied to verify the effectiveness of the latent class model adjustment. Clinical indicators were considered statistically significant if at least one of the confidence intervals (either for sensitivity or specificity) were above 0.5 (Collins and Lanza, 2010). Indicators that exhibited the worst performance for the area under the ROC curve were sequentially removed until the latent class model attained the proper fit. The area under ROC curves for dichotomous data is calculated based on the average sensitivity and specificity, namely (Se+Sp)/2. The adjusted model comprised the set of clinical indicators that presented the better best performance in terms of sensitivity and specificity to extract the latent variable structure.

*Ethical considerations*

The study was approved by the ethics committee of the university in which the study was undertaken, and all participants signed informed consents before participation.

## Results

*Population general data*

Of the 240 cancer patients undergoing chemotherapy, 83.8% were female, 83.8% were retirees, 81.3% had a religion, and 53.8% were married. The average age and length of education were 55.6 years (SD = 12.0) and 7.5 years (SD = 5.2).

The patients presented an average length of cancer diagnosis of 23.4 months (SD = 39.7), and the main form of cancer was breast cancer (43.8%). The most prevalent medications used were gastric and corticosteroid drugs (100%), antiemetic drugs (95.8%), and antiallergic drugs (40.8%).

*Clinical indicators of constipation*

The frequency of the clinical indicators of constipation is presented in Table 1. The most frequent clinical indicators were straining with defecation, decreased stool frequency, change in bowel pattern, hard formed stool, hypoactive bowel sounds, pain with defecation, and decreased stool volume. The following six clinical indicators were not included in the table mentioned above as they were absent: atypical presentations in older adults, borborygmi, distended abdomen, palpable abdominal mass, abdominal tenderness with palpable muscle resistance, and abdominal tenderness without palpable muscle resistance.

*Accuracy of clinical indicators of constipation*

All clinical indicators identified were included in the latent class analysis, forming the initial null model. Therefore, the indicators that presented undesirable results were sequentially excluded from the data set. Table 2 presents the adjusted latent class model.

Table 2 presents the set of clinical indicators capable of predicting constipation in cancer patients undergoing chemotherapy. The clinical indicators straining with defecation (100.0%) and hypoactive bowel sounds (75.0%) had high sensitivity values. On the other hand, the clinical indicators headache (99.9%), abdominal pain (75.0%), pain with defecation (75.0%), straining with defecation (99.9%), and liquid stool (78.1%) had high specificity values.

The clinical indicator straining with defecation was the most accurate in determining constipation in the patients studied as its presence was significantly associated with the presence of constipation, and its absence with the absence of this condition.

From the set of clinical indicators analyses together, it was possible to highlight the occurrence of the latent variable, constipation. The adjusted model presented a prevalence of constipation of 86.6% in the sample studied.

**Table 1:** Frequency of Clinical Indicators of the Nursing Diagnosis of Constipation in Cancer Patients Undergoing Chemotherapy

Clinical indicators	n	%
Straining with defecation	208	86.7
Decrease in stool frequency	208	86.7
Change in bowel pattern	192	80.0
Hard, formed stool	190	79.2
Hypoactive bowel sounds	171	71.3
Pain with defecation	139	57.9
Decrease in stool volume	135	56.3
Fatigue	117	48.8
Anorexia	116	48.3
Severe flatus	90	37.5
Hyperactive bowel sounds	69	28.8
Percussed abdominal dullness	56	23.3
Indigestion	55	22.9
Bright red blood with stool	44	18.3
Rectal fullness	35	14.6
Rectal pressure	35	14.6
Headache	34	14.2
Liquid stool	33	13.8
Vomiting	13	5.4
Abdominal pain	9	3.8

**Table 2:** Accuracy Measures of the Clinical Indicators of Constipation from the Adjusted Latent Class Model

Clinical Indicators	Se (%)	95% CI		Sp (%)	95% CI	
Headache	16.3	0.1192	0.2150	99.9	0.9998	10,000
Abdominal pain	0.4	0.0002	0.9768	75.0	0.5434	0.8764
Pain with defecation	62.9	0.5553	0.6930	75.0	0.1159	0.9709
Straining with defecation	100.0	0.9998	10,000	99.9	0.9448	10,000
Liquid stool	12.5	0.0910	0.1827	78.1	0.5750	0.8917
Hypoactive bowel sounds	75.0	0.6877	0.7992	53.1	0.3157	0.7184
Prevalence: 86.66%		G^2:^ 60.98		df: 50	p = 0.137	

Abbreviations,Se, sensitivity; Sp, specificity; CI, Confidence interval.

## Discussion

The present study found a high prevalence of constipation among females and breast cancer patients. These data corroborate the current scenario since breast cancer is the most common type of cancer in women (Brazil, 2018). 

The most used medications in the sample studied were gastric medications, corticosteroids, and antiemetic drugs. The drugs used in chemotherapy act on fast-growing cells, such as gastrointestinal, capillary, and immune cells. As a result, adverse effects can occur, such as nausea, vomiting, diarrhea, alopecia, and increased susceptibility to infections. Thus, the use of different pharmacological classes to treat undesirable symptoms like the ones mentioned above, is common (Silva and Comarella, 2013). 

Constipation was present in 86.6% of cancer patients in this study. The literature corroborates that constipation is the most frequent adverse effect in the population studied, especially in those receiving opioid analgesics or drugs with anticholinergic properties (Wickham, 2017). 

Constipation contributes to decreased quality of life in cancer patients. When left untreated, it results in great discomfort and negative consequences, some of which, life-threatening, such as bowel impaction and perforation (Woolery et al., 2008). Thus, nurses should use accurate clinical indicators to confirm the diagnosis of constipation and intervene in this problem promptly and based on evidence.

This study presents a set of six clinical indicators (straining with defecation, hypoactive bowel sounds, headache, abdominal pain, pain with defecation, and liquid stool) significantly associated with the occurrence of constipation in cancer patients undergoing chemotherapy. The literature confirms the relationship between constipation and the clinical indicators mentioned above (Emmanuel et al., 2017; Stubhaug, 2016). Therefore, the nurse should assess the patient’s history and perform a physical examination based mainly on accurate constipation indicators.

The clinical indicator straining with defecation can predict constipation accurately. The literature shows that straining with defecation, hardened stools, abdominal distension, and incomplete bowel movement are clinical manifestations of drug-induced constipation (Stubhaug, 2016). 

The clinical indicator hypoactive bowel sounds was considered sensitive for the detection of constipation. This clinical indicator often manifests itself together with hard and dry stools, which pass slowly through the intestines causing abdominal distension and a reduced frequency of bowel movements. As a result, uncomfortable and debilitating reactions that characterize constipation are experienced by the patient (Locasale et al., 2016).

Headache, which had a high specificity value, can be associated with gastrointestinal disorders (Martami et al., 2018). The brain and gut have a strong two-way connection via neural and immune pathways. This gut-brain axis plays an important role in the association between gastrointestinal disorders and headache (Mayer et al., 2011). Abdominal pain and pain with defecation were evidenced as specific clinical indicators. The literature notes pain as one of the main characteristics in these patients (Rhondali et al., 2013). Thus, it is relevant to accurately recognize constipation to relieve patients’ discomfort, providing adequate and comfortable bowel habits (Larkin et al., 2008; Wickham, 2017).

The clinical indicator liquid stools was specific for the detection of constipation. Episodes of liquid stools may occur as a consequence of prolonged constipation and paradoxical diarrhea. The mechanism occurs through the irritation of the rectal mucosa by the presence of fecaloma, leading to the production of a large amount of mucus, which resembles diarrheal stools (Wald, 2016). Nurses must correctly identify constipation in cancer patients and promote prompt and cost-effective interventions to the patient’s well-being. Dietary and lifestyle modification can be recommended by nurses (Toner and Claro, 2012) identifying accurate identification clinical indicators of constipation.

This study provides estimates of the accuracy of clinical indicators of constipation in patients undergoing chemotherapy that nurses can use to propose prompt and effective interventions. Therefore, this knowledge contributes to nursing science and enhances the clinical nursing practice in the cancer care field.

The limitations of this study were data collection conducted in only one center in Brazil and the adjustment in the latest model carried out to identify accurate indicators. Thus, multicenter accuracy studies on constipation are recommended to compare the present findings.

A high prevalence of constipation was found among cancer patients undergoing chemotherapy. The sensitive clinical indicators were straining with defecation and hypoactive bowel sounds. The indicators headache, abdominal pain, pain with defecation, straining with defecation, and liquid stool were specific. The indicator straining with defecation was considered a reliable predictor of constipation. In conclusion, nurse practitioners should identify sensitive and specific clinical indicators to confirm chronic constipation in cancer patients undergoing chemotherapy.
